# Kinetics of dried blood spot-measured anti-SARS-CoV2 Spike IgG in mRNA-vaccinated healthcare workers

**DOI:** 10.3389/fmicb.2023.1130677

**Published:** 2023-03-01

**Authors:** Lucrezia Puccini, Michela Fantini, Carlo Biagetti, Raffaella Angelini, Giorgio Dirani, Laura Grumiro, Pasqua Schiavone, Monica Sparacino, Simona Semprini, Vittorio Sambri, Monica Cricca

**Affiliations:** ^1^Operative Unit of Microbiology, The Great Romagna Hub Laboratory, Pievesestina, Italy; ^2^Health Services Research, Evaluation and Policy Unit, AUSL Romagna, Rimini, Italy; ^3^Operative Unit of Infectious Disease, Ospedale Infermi, Rimini, Italy; ^4^Public Health Department, Ravenna, Italy; ^5^Department of Experimental, Diagnostic and Specialty Medicine-DIMES, University of Bologna, Bologna, Italy

**Keywords:** COVID-19, SARS-CoV2 IgG, dried blood spot, mRNA vaccine, booster

## Abstract

**Introduction:**

One of the major criticisms facing the research community during SARS-CoV2 pandemic was the lack of large-scale, longitudinal data on the efficacy of the SARS-CoV2 mRNA vaccines. Currently, even if COVID-19 antiviral treatments have been authorized by European Medicine Agency, prevention through approved specific vaccines is the best approach available in order to contain the ongoing pandemic.

**Objectives:**

Here, we studied the antibody kinetic over a one-year period from vaccination with the Pfizer-BioNTech (Pfizer) vaccines and subsequent boosting with either the BioNTech or Moderna (Spikevax) vaccines in a large cohort of 8,071 healthcare workers (HCW). We also described the impact of SARS-CoV2 infection on antibody kinetic over the same period.

**Methods:**

We assessed the *anti SARS*-*CoV2* Spike *IgG antibody* kinetic by the high throughput dried blood spot (DBS) collection method and the GSP®/DELFIA® Anti-SARS-CoV2 IgG assay (PerkinElmer®).

**Results:**

Our data support existing models showing that SARS-CoV2 vaccination elicits strong initial antibodies responses that decline with time but are transitorily increased by administering a vaccine booster. We also showed that using heterologous vaccine/booster combinations a stronger antibody response was elicited than utilizing a booster from the same vaccine manufacturer. Furthermore, by considering the impact of SARS-CoV2 infection occurrence in proximity to the scheduled booster administration, we confirmed that booster dose did not contribute significantly to elicit higher antibody responses.

**Conclusion:**

DBS sampling in our large population of HCWs was fundamental to collect a large number of specimens and to clarify the effective mRNA vaccine-induced antibody kinetic and the role of both heterologous boosters and SARS-CoV2 infection in modulating antibody responses.

## Introduction

Coronavirus disease 2019 (COVID-19) is a new zoonotic respiratory disease caused by severe acute respiratory syndrome coronavirus 2 (SARS-CoV2), appeared at the end of 2019 and responsible for the actual global pandemic, that to date is accounting for 635.105.062 cases and 6.609.981deaths worldwide (data revised on 13/11/2022) (Johns Hopkins University and Medicine, n.d.; Polat and Ergunay, 2021). Since the beginning of the pandemic, the main aim was to achieve prevention through the development of specific vaccines, leading to host production of antibodies directed toward the Receptor Binding Domain (RBD) of SARS-CoV2 spike (S) protein, preventing attachment to the host cell and neutralizing the virus. To date, precise characterization of the human antibody response to SARS-CoV2 infection and vaccination is not clearly defined yet and it would be vitally important to better define vaccine’s protection durability, even if the contribution of both humoral and cellular response must be considered (Kalimuddin et al., 2021). Once defined, specific antibody kinetic will become increasingly important in planning eventual additional doses of vaccine and powering efficacy studies. Also, reports of waning vaccine efficacy, coupled with the emergence of variants of concern (VOCs) that are resistant to antibody neutralization, have raised concerns about the potential lack of durability of immunity to vaccination (Hoffmann et al., 2021). However, little is known about T-cell cross reactivity with VOCs (Alsobaie, 2021; Geers et al., 2021). So, even if humoral response alone is not enough to explain the whole different immune responses combinations, infection-or vaccine-induced antibody levels are a useful indicator of a vaccination campaign performance (Khoury et al., 2021), considering that different studies demonstrated a good correlation between established antibody levels and vaccine efficacy against several VOCs (Amanat et al., 2020; Tran et al., 2022). In this sense, establishing antibody titres and lifetime as a correlate of protection and defining a protective cut-off should be priorities to investigate protection provided by natural infection or vaccination (Khoury et al., 2021). Beside evaluating vaccine efficacy, monitoring immunoglobulin levels across a broader temporal spectrum is fundamental to understand their kinetics from the beginning of infection onwards, defining IgG persistence time (Alharbi et al., 2022).

Humoral immune responses to the SARS-CoV2 S protein are typically evaluated by Enzyme Linked Immunosorbent Assay (ELISA) and its many variants (CLIA, LFA, etc.) (Bagno et al., 2022). However, conventional laboratory testing on plasma or serum samples is not always readily available. Because of this, an alternative collection method has been introduced for serological testing, Dried Blood Spot (DBS) collection, where a sample of capillary blood is obtained from few blood drops taken from patient’s fingertip (Tuaillon et al., 2020). DBS samples are easily collected and stored, also at a distance, and transported by mail, facilitating more widespread testing by overcoming some relevant obstacles and facilitating collection in different settings. Also, follow up both in case of infection or to monitor antibodies’ kinetic after vaccination would be easier (Brinc et al., 2021). Indeed, the feasibility of DBS serology testing to detect specific antibodies can allow for widespread monitoring of vaccine responses (Montesinos et al., 2021).

## Materials and methods

### Study design and population

A total of 8,071 health care workers (HCWs), from the Great Romagna Area, Emilia-Romagna region, Italy, were enrolled in this study from February 2021 to March 2022 to evaluate the anti SARS-CoV2 IgG serological kinetic after primary anti SARS-CoV2 mRNA vaccination and vaccine booster. The study protocol was approved by the Local Ethics Committee (protocol number: 946/2021 SIEROVAC) and all the HCWs provided written informed consent. No significant comorbidities known to affect vaccine efficacy were reported. A total of 1,962 men (24%) and 6,109 women (76%), with a mean age of 47.2 (CI 46.5–47.7) and 46.32 (CI 46.1–48.6), respectively, were enrolled in this study. Detailed demographic characteristics are reported in Table 1.

**Table 1:** Demographic characteristics of the HCWs.

Age groups	Male (*N*)	Female (*N*)	Male (Mean age, CI 95%)	Female (Mean age, CI 95%)
<40	627	1,887	33.6 (33.3–33.9)	32.27 (32.1–32.5)
40–55	771	2,704	48.45 (48.2–48.8)	48.79 (48.6–48.9)
>55	564	1,518	60.54 (60.3–60.8)	59.35 (59.2–59.5)
Total	1962	6,109	47.2 (46.5–47.7)	46.32 (46.1–48.6)

HCWs were recruited on a voluntary basis and all of them received the first cycle of vaccination (first and second doses scheduled at day 1 and 21) with Comirnaty vaccine (Pfizer/BioNTech) and homologous (64%) or heterologous (36%) boosters, Comirnaty and Spikevax (Moderna), respectively.

Then, HCWs were tested for anti-spike IgG antibodies at 5 time points (45, 90, 180, 270, and 365 days) after the first dose of vaccine, over a period of 1 year. A total of 7,757 HCWs (96%) received the booster, which in general was administered before the 5th determination of anti-spike IgG antibodies.

A total of 772 (9.6%) HCWs got the SARS-CoV2 infection before the 5th blood sampling (BS), more precisely, 423 (55%) got the infection before the 1st vaccine dose. No data are available about specific SARS-CoV2 variants among the population of SARS-CoV2 infected HCWs. According to local guidelines, since December 2020 and throughout all the study period, all the HCWs have been monitored every 3 weeks for SARS-CoV2 RNA by the Allplex 2019-nCoV Assay, Seegene, Seoul-South Corea, on nasopharyngeal swabs. We considered as infected, symptomatic or asymptomatic HCWs who tested positive for the molecular test.

### Specimen collection and anti-SARS CoV2 Spike IgG testing

DBS collection may be a practical testing solution owing to this method’s simplicity. DBS testing was carried out onto a collection card (226 Spot Saver Card, PerkinElmer) with sterile lancets by puncturing fingertip skin. Upon collection, DBS were allowed to dry for approximately 3 to 4 h at room temperature and then delivered to the laboratory, where they were stored at −20°C and analyzed within 1 week. The day before the analysis, DBS were thawed at +4°C and then analyzed after reaching room temperature.

Analysis of DBS was performed at the Microbiology Unit, Great Romagna Area Hub Laboratory, Pievesestina (FC), Italy, by using GSP®/DELFIA® Anti-SARS-CoV2 IgG assay (PerkinElmer®). GSP/DELFIA system was previously assessed in comparison with other 10 commercial assay by using DBS collection method, leading to an almost perfect agreement with the reference method (EURIMMUN, Lübeck, Germany) (Morley et al., 2020; Cholette et al., 2021; Turgeon et al., 2021). Briefly, one blood disk for each card was automatically punched into an assay well of a 96 well microtitre plate, coated with SARS-CoV2 Spike protein S1 subunit. Human anti-SARS-CoV2 IgG were eluted from the blood disk in order to react with the immobilized S1-antigens and then with the europium labeled anti-human IgG antibodies. Finally, the Dissociation-Enhanced Lanthanide Fluorescence ImmunoAssay (DELFIA) Inducer was added to dissociate europium ions from the labeled antibody into solution where they form highly fluorescent chelates which are measured by the instrument (Genetic Screening Processor, PerkinElmer®). In each run were analyzed one calibrator, one positive and one negative controls in duplicate. The results are given as ratios by dividing the sample signal by the average signal of the calibrator (300 ng/ml) provided in the kit. The cut-off value for anti-SARS-CoV2 IgG in DBS is 1.4, as determined by the manufacturer. In each run one positive and one negative control were analyzed in duplicate (Cicalini et al., 2022).

### Statistical analysis

Statistical analysis was performed with Stata version 17.0; StataCorp. Quantitative variables are expressed as mean and relative confidence interval (CI). When comparing mean values of two groups, we used *t*-test, while in comparisons between more groups we used ANOVA test, *p* value <0.001 was considered significant.

## Results

### Classification of HCWs in different groups based on SARS-CoV2 infection timing

A total of 8,071 HCWs were recruited for the study. Only 4,227 (52%) HCWs underwent all the five scheduled blood samplings (BS), while the remaining did at least one BS. The mean intervals between the first dose of vaccine and each of the five scheduled BS were as follow: 52 (SD = 5), 94 (SD = 5), 183 (SD = 4), 272 (*SD* = 5) and 368 (*SD* = 5) days, respectively.

Based on the fact that 771 (9.5%) HCWs got the SARS-CoV2 infection at different time points with respect to primary vaccination, we identified three different groups of HCWs: Group 1 (G1) included HCWs who never got the SARS-CoV2 infection (*n* = 7,299); Group 2 (G2) included HCWs who got the infection before the first dose of vaccine (*n* = 423), with a mean of (−)156 days (SD = 109); Group 3 (G3) included HCWs who got infected after the first dose of vaccine (*n* = 349), with a mean of (+) 209 days (SD = 149). Mean values and CI of anti-SARS-CoV2 IgG for each group and the five BSs are reported in Table 2.

**Table 2:** Mean values and CI of anti-SARS-CoV2 IgG for G1, G2, and G3 HCWs groups.

Sampling timing (Mean days 1st vaccine dose)		1st BS (52)	2nd BS (94)	3rd BS (183)	4th BS (272)	5th BS (368)
G1 (*N* = 7299 HCWs)	*N* (%)	7,238 (99%)	5,850 (80%)	5,839 (80%)	5,562 (76%)	4,227 (58%)
	Mean value (CI)	34.69 (34.26 – 35.14)	19.25 ss(18.91 – 19.59)	7.19 (6.99 – 7.38)	6.58 (6.26 – 6.91)	47.26 (46.58 – 47.95)
G2 (*N* = 423 HCWs)	*N* (%)	421 (100%)	348 (82%)	333 (79%)	326 (77%)	259 (61%)
	Mean value (CI)	48.80 (46.92 – 50.68)	45.47 (42.84 – 48.11)	26.42 (23.82 – 29.02)	23.87 (21.21 – 26.53)	51.23 (48.63 – 53.84)
G3 (*N* = 349 HCWs)	*N* (%)	348 (100%)	299 (86%)	302 (87%)	289 (82%)	276 (79%)
	Mean value (CI)	25.87 (23.93 – 27.81)	16.22 (14.71 – 17.73)	9.24 (7.62 – 10.85)	26.85 (22.62 – 31.07)	63.03 (60.48 – 65.59)

Stratifying data for age and sex in G1 group, we found significantly higher antibody level in people <40 years and in female vs. male for 1st and 2nd BSs. Furthermore, significant differences are present in people <40 years versus older for 3rd and 4th BSs and no significant differences in 5th BS (Supplementary Table S1).

To better describe G3 population, we further identified 5 different subgroups (SG) based on the timing of SARS-CoV2 infection with respect to the 5 scheduled BS (Table 3).

**Table 3:** G3 subgroups based on SARS-CoV2 timing of infection over a 1-year period after vaccination.

SG	SG4 (COVID-19 before the 1st BS)	SG5 (COVID-19 before the 2nd BS)	SG6 (COVID-19 before the 3rd BS)	SG7 (COVID-19 before the 4th BS)	SG8 (COVID-19 before the 5th BS)
*N* (%)	101 (29%)	13 (3%)	21 (6%)	34 (10%)	180 (52%)

SG, Subgroup; BS, blood sampling.

**Figure 1:**
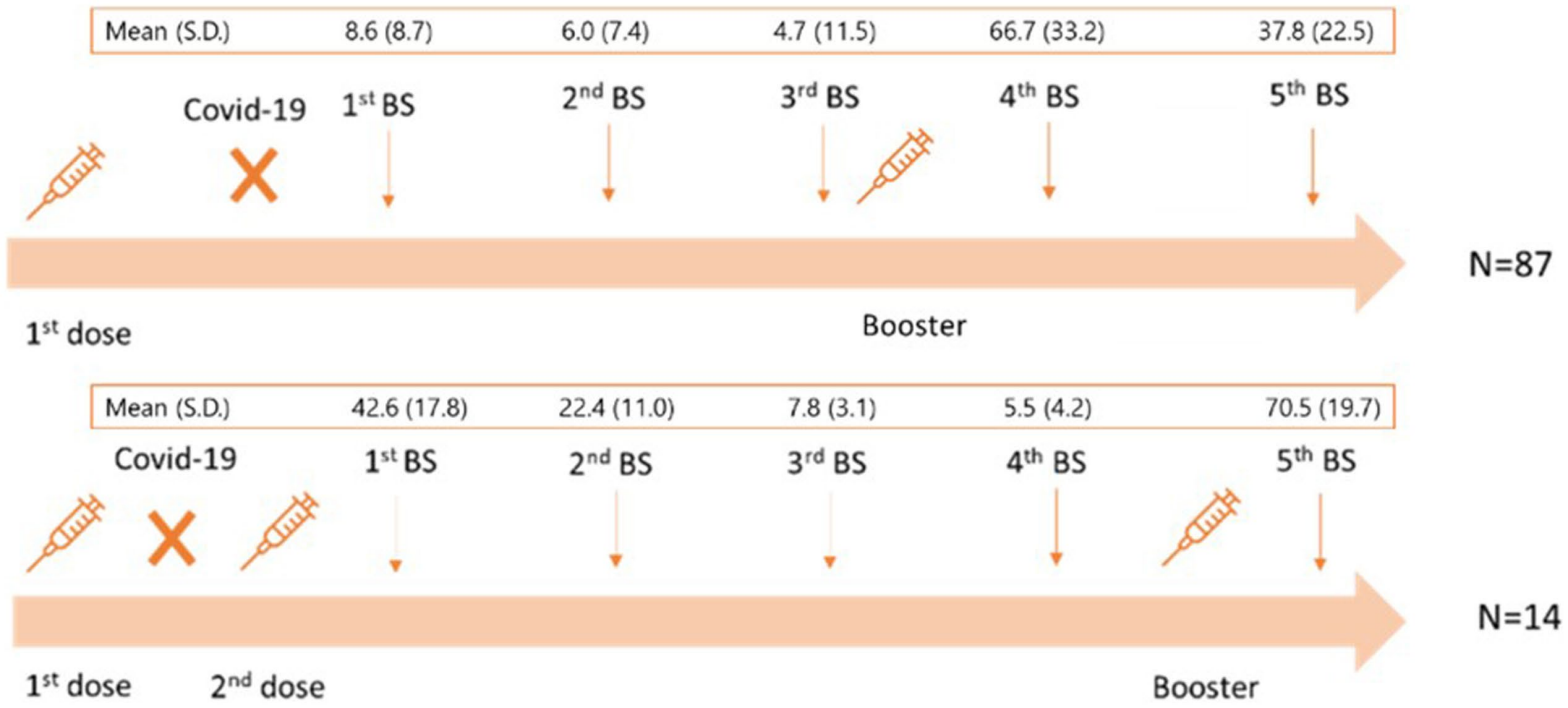
Schematic representation of SG4 subgroup. SG4: subgroup 4, SD: standard deviation.

We further analyzed subgroups 4 (SG4) and 8 (SG8), due to the greater number of cases. Among SG4 (Table 4), we observed a lower titre of anti-SARS-CoV2 IgG (13.35, CI = 10.26–16.45) of the 1st BS with respect to G1 HCWs (34.69, CI = 34.26–35.14). A total of 87 HCWs received a delayed 2nd vaccine dose (247 days, SD = 35), because the SARS-CoV2 infection replaced the administration of the 2nd vaccine dose (Figure 1). Their 1st BS antibody titre was significantly lower (8.6; CI = 6.8–10.5) than the remaining 14 HCWs who received the 2nd dose soon after infection (42.6; CI = 33.3–52.0) (*p* < 0.001) (Figure 1).

**Table 4:** Mean values and C.I. of anti-SARS-CoV2 IgG for G4 group.

SG4 (*N* = 101)	1st BS	2snd BS	3rd BS	4th BS	5th BS
*N*	101 (100%)	74 (73%)	71 (70%)	62 (61%)	46 (46%)
Mean value	13.35	7.99	5.11	56.37	42.82
C.I.	10.26–16.45	5.80–10.20	2.53–7.69	46.83–65.92	35.52–50.11

BS, blood sampling; C.I., confidence interval, SG4, HCWs who got the COVID-19 before the 1st BS.

**Figure 2:**
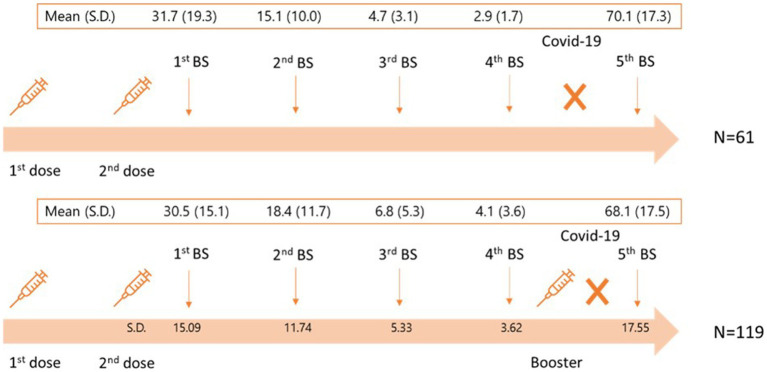
Schematic representation of SG8 subgroup. SG8: subgroup 8, SD: standard deviation.

Subgroup 8 (SG8) (Table 5) showed a mean interval of 340 days (SD = 30) and 35 days (SD = 27.5) from the 1st vaccine dose to SARS-CoV2 infection and from infection to 5th BS, respectively. In this subgroup, 119 subjects got the COVID-19 after the booster dose and about 20 days before the last scheduled BS, while 61 HCWs did not receive the booster dose because of the infection timing (59 days before the 5th BS) (Figure 2). We did not observe any significant difference in antibody titre among the two subpopulations (68.3 vs. 70.1; *p* = 0.514) despite the booster.

**Table 5:** Mean values and C.I. of anti-SARS-CoV2 IgG for G8 group.

SG8 (*N* = 180)	1st BS	2nd BS	3rd BS	4th BS	5th BS
*N*	180 (100%)	169 (94%)	168 (93%)	164 (91%)	180 (100%)
Mean value	30.94	17.35	6.10	3.76	68.90
C.I.	28.50–33.38	15.64–19.06	5.37–6.83	3.27–4.25	66.33–71.46

BS, blood sampling; C.I., confidence interval; SG8, HCWs who got the COVID-19 before the 5th BS.

### Antibody kinetics

**Figure 3:**
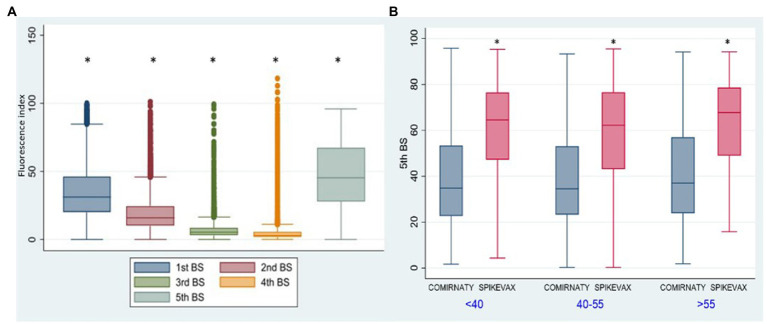
Group 1: box plot of anti-SARSCoV-2 antibody kinetics. *p < 0.001. Fluorescence index: sample signal by the average signal of the calibrator 5th BS: IgG anti-SARS-CoV-2 fluorescent index of the fifth blood sampling. <40: age <40 y.o., 40-55: age from 40 to 55 y.o. >55: age >50 y.o. Comirnaty: Comirnaty mRNA booster, Spikevax: Spikevax mRNA booster.

Antibody kinetics of G1 (Figure 3A) showed a gradual decline in antibody titre from the 1st to the 4th BS and an increase in the 5th BS due to the booster dose (*p* < 0.001). Thirty-six percent of HCWs received the Spikevax vaccine as booster dose, while 64% received the Comirnaty. The antibody titre of the 5th BS (60.66 vs. 39.68) was significantly (*p* < 0.001) higher utilizing the Spikevax booster (Figure 3B).

**Figure 4:**
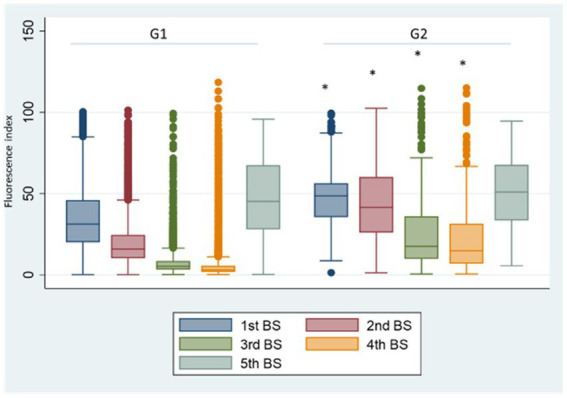
Group 1: box plot of BS values at different samplings. *p < 0.001, Fluorescence index: sample signal by the average signal of the calibrator, G1: group 1, G2: group 2.

In G2 (Figure 4) we observed a significant increase (p < 0.001) of anti SARS-CoV2 antibody titres for the 1st, 2nd, 3rd, and 4th BSs vs. the corresponding BSs of G1 group, due to the contribution of natural immunity before the 1st dose of vaccine. While no significant difference was present between the two groups for the 5th BS, due to the booster dose.

**Figure 5:**
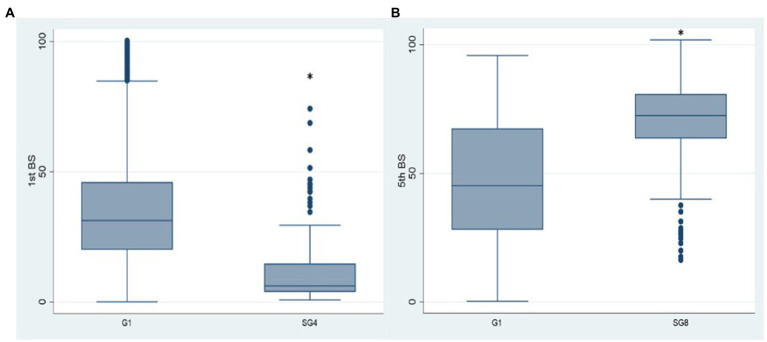
Box plot of the antibody values of the 1st and 5th BSs in the SG4 (A) and SG8 (B) groups, respectively. *p < 0.001, BS: blood sampling, G1: group 1, SG4: subgroup 4, SG8: subgroup 8.

Comparing SG4 antibody levels with G1 (Figure 5A), the 1st BS titre is significantly lower than that referred to G1 (*p* < 0.001). The reason was that the natural infection replaced the 2nd vaccine dose in the majority of the HCWs of SG4 leading to an incomplete primary vaccination cycle administration. In SG8 (Figure 5B), the antibody titre of the 5th BS is significantly higher than the one referred to G1 (68.90 vs. 47.26; *p* < 0.001) underpinning the role of natural infection in eliciting antibody response.

## Discussion

In this study, we aimed to evaluate antibody kinetics of anti SARS-CoV2 IgG antibodies in one of the largest cohort of healthcare workers to date over a 1-year period after primary vaccination cycle with BioNTech mRNA vaccine. We also evaluate the impact of homologous (BioNTech) and heterologous (Spikevax) boosters and the role of SARS-CoV2 infection in the modulation of anti S1 SARS-CoV2 IgG response. DBS specimens were reliably used as an alternative to serum samples for SARS-CoV-2 antibody measurement. DBS sampling was crucial for the enrolment of a such large cohort of HCW, facilitating collection and storage of specimens.

This study was conducted during a period of high prevalence of the Delta variant (from March 2021), and shortly after the emergence of the Omicron one. The study was based on vaccine antibody response, without testing for IgG neutralization potential. Some possible limitations of the study are that only humoral immune responses is considered. As reported elsewhere, the effectiveness of memory B and T cells may be important for long-term protection (Gianfagna et al., 2022). Furthermore, we did not identify a protection cut-off.

Our data strengthen previous findings demonstrating that immunization to SARS-CoV2 through vaccination induces a peak in antibody response soon after vaccination, followed by a marked and progressive decline of anti-Spike IgG antibodies during a 9 months period after vaccination (Levin et al., 2021; Chivu-Economescu et al., 2022).

Also, we found that in naïve individuals, younger age (<40 years) and female sex are associated with higher antibody levels for the 1st and 2nd BS, with this age-related difference observed up to the 4th BS. The antibody response to the third dose was different in that a significant inverse relationship was not detected between age, sex and antibody levels, confirming literature data (Notarte et al., 2021; Tanaka et al., 2022).

The booster dose induced a transitory increase in humoral response, restoring antibody levels to the initial values yielded by primary vaccination (Chivu-Economescu et al., 2022; Desmecht et al., 2022), particularly in naïve individuals (never infected). Even more, heterologous vaccination (primary vaccination with Comirnaty vaccine followed by Spikevax booster) elicited higher anti-SARS-CoV2 antibodies response with respect to homologous (Comirnaty/Comirnaty) vaccination regimens, confirming literature data (Naito et al., 2022).

Also, we analyzed the role of natural infection in the modulation of anti-SARS-CoV2 antibody kinetics. In a recent study (Cholette et al., 2021), SARS-CoV2 infection before vaccination was significantly associated with higher antibody titres up to 6 months after infection. Accordingly, we confirmed a greater IgG anti SARS-CoV2 titre in HCWs who got the infection before full primary vaccination (G2 group), underlining the contribution of natural immunization in enhancing the antibody response in non-naïve patients versus the naïve ones (Costa et al., 2022). Furthermore, we observed higher anti SARS-CoV2 levels in naïve HCWs who underwent a complete primary vaccination cycle with respect to HCWs whose second dose was replaced by natural infections, underpinning the importance of accomplishing the primary vaccination course.

Our data highlight no significant difference in anti-SARS-CoV2 antibody levels in HCWs who received the booster before the 5th BS and those who got the infection soon after the booster (SG8), conversely to what is reported in other studies (Chivu-Economescu et al., 2022). Also, we observed higher antibody titres in individuals who additionally got the infection before the 5th BS compared to naïve HCWs (SG8 vs. G1), highlighting the greater impact of natural immunization on antibody titres after the complete primary vaccination cycle, with respect to the booster dose.

In conclusion, we confirmed a progressive decline in antibody levels until 9 months after Comirnaty vaccination and an increase titre after the booster administration. Heterologous boosters with Spikevax significantly enhanced the antibody titre when compared to homologous regimens. We also highlight the role of natural immunization in the modulation of antibody kinetics.

## Data availability statement

The original contributions presented in the study are included in the article/Supplementary material, further inquiries can be directed to the corresponding author.

## Ethics statement

The studies involving human participants were reviewed and approved by CEROM, AUSL ROMAGNA. The patients/participants provided their written informed consent to participate in this study.

## Author contributions

LP contributed to data analysis, and writing. MF contributed to statistical analysis. CB contributed to data analysis. RA contributed to the study design. GD, LG, PS, and MS discussed the results and commented on the manuscript. SS contributed to study design, data analysis, and commented on the manuscript. VS contributed to the study design, discussed the results, and commented on the manuscript. MC discussed the results and contributed to data analysis and writing. All authors contributed to the article and approved the submitted version.

## Funding

This study was supported by “Fondo per la Ricerca e l’Innovazione, Area Vasta della Romagna,” Emilia-Romagna, Italy, independently of study sponsors. Perkin Elmer supported this study with consumables and equipments. We are grateful to Morotti L., Mambelli S., Cappucci S., Delvecchio F., Esposito L., Leoni G., Marchini B., Mazzotti L., Romano R., Sacconi A., Sternini V., Scatasta G., Ceccarelli B., Coltraro M., Dal Re S., for technical support.

## Conflict of interest

The authors declare that the research was conducted in the absence of any commercial or financial relationships that could be construed as a potential conflict of interest.

## Publisher’s note

All claims expressed in this article are solely those of the authors and do not necessarily represent those of their affiliated organizations, or those of the publisher, the editors and the reviewers. Any product that may be evaluated in this article, or claim that may be made by its manufacturer, is not guaranteed or endorsed by the publisher.
