# Supplementary material for: Kinetics of dried blood spot-measured anti-SARS-CoV2 Spike IgG in mRNA-vaccinated healthcare workers
Source: Front Microbiol. 2023 Mar 1;14:1130677. doi: 10.3389/fmicb.2023.1130677 (PMC10014594; doi:10.3389/fmicb.2023.1130677)
Supplement: Supplementary file 1 [file Table-2.docx]

**Supplemental Table**

Supplemental Table 1 (Table S1)

Table S1: distribution according to sex and age considering the 5 BSs, mean and CI are reported for each category.

| **Age groups/Sampling timing** | | **1^st^ BS**  **mean (DS)** | **2^nd^ BS**  **mean (DS)** | **3^rd^ BS**  **mean (DS)** | | **4^th^ BS**  **mean (DS)** | | **5^th^ BS**  **mean (DS)** | |  |  |
| --- | --- | --- | --- | --- | --- | --- | --- | --- | --- | --- | --- |
| **G1** | <40 | 37.2 (19.0) | 21.8 (14.4) | 9.0 (7.8) | | 7.3 (11.7) | | 50.0 (20.9) | |  |  |
| Male | 40-55 | 31.7 (18.4) | 16.8 (12.2) | 6.9 (8.3) | | 6.1 (11.6) | | 47.2 (22.7) | |  |  |
|  | >55 | 27.4 (17.6) | 15.2 (12.0) | 6.1 (8.3) | | 7.2 (15.2) | | 44.3 (23.3) | |  |  |
| **G1** | <40 | 39.5 (19.4) | 22.4 (13.8) | 8.3 (6.9) | | 7.5 (12.3) | | 47.5 (23.1) | |  |  |
| Female | 40-55 | 33.8 (18.5) | 18.2 (12.2) | 6.3 (6.1) | | 5.9 (11.2) | | 46.2 (22.6) | |  |  |
|  | >55 | 33.8 (19.4) | 19.4 (14.2) | 7.5 (8.8) | | 6.7 (13.1) | | 49.0 (23.1) | |  |  |
| **G2** | <40 | 48.6 (20.6) | 51.0 (26.3) | 23.6 (17.5) | | 20.6 (16.7) | | 56.3 (17.4) | |  |  |
| Male | 40-55 | 50.9 (19.8) | 51.4 (24.1) | 32.0 (27.4) | | 28.3 (27.2) | | 54.6 (21.9) | |  |  |
|  | >55 | 45.5 (20.6) | 51.0 (26.3) | 40.4 (35.6) | | 43.2 (39.5) | | 53.1 (26.4) | |  |  |
| **G2** | <40 | 45.9 (17.8) | 40.1 (22.8) | 20.7 (17.0) | | 17.7 (16.6) | | 49.3 (19.3) | |  |  |
| Female | 40-55 | 49.0 (21.4) | 43.3 (24.2) | 24.2 (22.6) | | 20.6 (21.6) | | 46.5 (21.2) | |  |  |
|  | >55 | 55.1 (17.3) | 52.8 (26.2) | 34.2 (29.6) | | 32.5 (28.2) | | 59.6 (20.8) | |  |  |
| **G3** | <40 | 19.2 (20.1) | 11.2 (12.3) | 15.2 (4.0) | | 26.2 (41.8) | | 58.8 (24.1) | |  |  |
| Male | 40-55 | 21.4 (18.6) | 14.3 (12.4) | 6.5 (8.3) | | 23.3 (35.7) | | 59.7 (22.8) | |  |  |
|  | >55 | 27.2 (20.1) | 16.8 (12.3) | 6.5 (4.0) | | 32.3 (41.9) | | 66.2 (24.1) | |  |  |
| **G3** | <40 | 28.7 (20.3) | 17.0 (13.7) | 9.2 (13.6) | | 29.4 (37.5) | | 64.7 (20.2) | |  |  |
| Female | 40-55 | 26.9 (18.9) | 17.0 (13.6) | 9.1 (13.5) | | 26.8 (36.1) | | 60.8 (22.7) | |  |  |
|  | >55 | 24.2 (15.1) | 16.0 (13.6) | 10.0 (16.9) | | 23.8 (37.6) | | 68.1 (18.4) | |  |  |
|  |  |  | | |  | |  | |  | |  |

BS: blood sampling; CI: confidence interval; HCWs: Health Care -workers; G1: HCWs who never got the SARS-CoV2 infection; G2: HCWs who got the SARS-CoV2 infection before the 1^st^ dose of mRNA vaccine; G3: HCWs who got the SARS-CoV2 infection after the 1^st^ dose of mRNA vaccine.
